# Trends in mortality rates by subtypes of heart disease in Mississippi, 1980–2013

**DOI:** 10.1186/s12872-017-0593-3

**Published:** 2017-06-15

**Authors:** Vincent L. Mendy, Rodolfo Vargas, Marinelle Payton

**Affiliations:** 1 Office of Health Data and Research, Mississippi State Department of Health, 570 East Woodrow Wilson, Jackson, MS 39215 USA; 2 Center of Excellence in Minority Health and Health Disparities, Institute of Epidemiology and Health Services Research, School of Public Health, Jackson State University, Jackson, USA

**Keywords:** Average annual percent change, Heart disease subtypes, Mortality, Mississippi, Trends

## Abstract

**Background:**

Heart disease (HD) is the leading cause of death among Mississippians. However, trends in mortality rates for HD subtypes in Mississippi have not been adequately described. This study examined trends in mortality rates for HD subtypes among adults in Mississippi from 1980 through 2013.

**Methods:**

We used Mississippi Vital Statistics data to calculate age-specific mortality rates for HD subtypes for Mississippians age 35 and older. Cases were identified via underlying cause of death codes from the International Classification of Diseases, Ninth Revision (ICD-9) and Tenth Revision (ICD-10). We used Joinpoint software to calculate the average annual percent change (AAPC) in mortality rates for HD subtypes by race, sex, and age group.

**Results:**

Overall, the age-adjusted coronary heart disease (CHD) mortality rate among Mississippi adults decreased by 62.7% between 1980 and 2013, with an AAPC of −3.0% (95% CI −3.7 to −2.3), while the age-adjusted heart failure mortality rate increased by 66.7%, with an AAPC of 1.4% (95% CI 0.5 to 2.3). Trends varied across HD subtypes: Annual rates of hypertensive HD mortality increased significantly for men, for individuals age 35 to 54, and for individuals age 75 and older. CHD mortality experienced a significant annual decrease among all race, sex, and age subgroups, while heart failure increased significantly among women, whites, and individuals age 75 and older.

**Conclusions:**

From 1980 to 2013, CHD mortality decreased significantly while heart failure mortality increased significantly among adult Mississippians. However, HD subtype trends differed by race, sex, and age group.

## Background

Heart disease (HD) is the leading cause of death among adults in the United States (U.S.) [1]. In 2013, the HD mortality rate in Mississippi was 1.4 times higher than the national rate [2]. HD accounted for a quarter of all deaths among Mississippi adults in 2013, and an even larger proportion of deaths among blacks and men in the state [3].

A recent national study indicated that HD mortality declined substantially among U.S. adults from 1973 and 2010, although the pattern of decline varied by race and geography [4]. Researchers have attributed these declines to both reductions in major risk factors for HD (e.g., cigarette smoking, hypertension, and hyperlipidemia) and the use of evidence-based medical and pharmaceutical therapies that improve disease control (e.g., individuals with hypertension managing their blood pressure) [5, 6].

While there is extensive information on overall HD trends, there is a dearth of information on trends for HD subtypes, both nationally [3, 4, 7] and in Mississippi. Specifically, there is limited information on trends in mortality rates for HD subtypes in Mississippi. To address this gap, we calculated the AAPC in age-adjusted mortality rates for HD subtypes among Mississippi adults (≥35 years of age) between 1980 and 2013. In addition, we examined how AAPC varied by race, sex, and age group.

## Methods

The number of adult (≥35 years of age) deaths due to HD for each year from 1980 through 2013 were extracted from Mississippi Vital Statistics [3]. We used the underlying cause of death codes from the International Classification of Diseases, Ninth Revision (ICD-9) and Tenth Revision (ICD-10) for hypertensive HD [ICD-9:402; ICD-10:I11,I13], coronary heart disease (CHD) [ICD-9: 402, 410–414,429.2; ICD-10: I20-I25], and heart failure [ICD-9:428; ICD-10: I50] [7]; ICD-9 was used for data from 1980 through 1999 and ICD-10 was used for data from later years. Other HD subtypes (acute rheumatic fever, chronic rheumatic heart disease, pulmonary heart disease) were not included because the data were insufficient. We then used Mississippi population census counts to calculate the age-adjusted HD mortality rates and standard errors for the state as a whole, for race subgroups (blacks, whites), and for gender subgroups (women, men). Age-adjustment was conducted via the direct method, based on the 2000 U.S. standard projected population [8].

Next, we exported age-adjusted mortality rates for HD subtypes and standard errors to the U.S. Surveillance, Epidemiology, and End Results (SEER) Joinpoint software (4.1.1.5) (http://surveillance.cancer.gov/joinpoint/) to calculate the AAPC in HD mortality rates by race, sex, and age group. Joinpoint regression analysis identifies trend breaks (joinpoints)—points of significant change in a trend [7, 9]. This analysis identified time periods with statistically distinct log-linear trends in mortality rates for each HD subtype [9]. Using a Bayesian information criterion approach to select the most parsimonious model of best fit, we specified a maximum of three joinpoints [8–11]. The slopes of the models were used to calculate the average percentage change (APC) for each trend segment and the AAPC (the weighted average of the APC) [7]. For each AAPC, 95% confidence intervals (CIs) were calculated and tested to determine whether the change was statistically significant (significantly different from the null hypothesis of no change, or 0%) [12, 13] using a *p*-value of <0.05. This investigation was approved by the Mississippi State Department of Health Institutional Review Board.

## Results

Overall, from 1980 through 2013, the age-adjusted CHD mortality rate decreased by 62.7%, and the AAPC was −3.0% (95% CI −3.7 to −2.3). For heart failure, the age-adjusted mortality rate increased by 66.7%, and the AAPC was 1.4% (95% CI 0.5 to 2.3).

Age-adjusted mortality rates for subtypes of HD varied by race, sex, and age group.

CHD mortality rates decreased significantly for all subgroups, with an AAPC of −3.0% (95% CI −3.6 to −2.3) for men, −3.1% (95% CI −3.6 to −2.5) for women, −3.1% (95% CI −3.4 to −2.7) for whites, −3.1% (95% CI −4.2 to −2.0) for blacks, −1.5% (95% CI −1.7 to −1.2) for individuals age 35–54, −3.0% (95% CI −3.6 to −2.3) for individuals age 55–74, and −3.2% (95% CI −3.7 to −2.8) for individuals age 75 and older.

In contrast, hypertensive HD mortality rates increased significantly among certain subgroups, with an AAPC of 1.6% (95% CI 0.3 to 3.0) for men, 4.5% (3.6 to 5.5) for individuals age 35–54, and 1.4% (95% CI 0.2 to 2.6) for individuals age 75 and older.

**Fig. 1:**
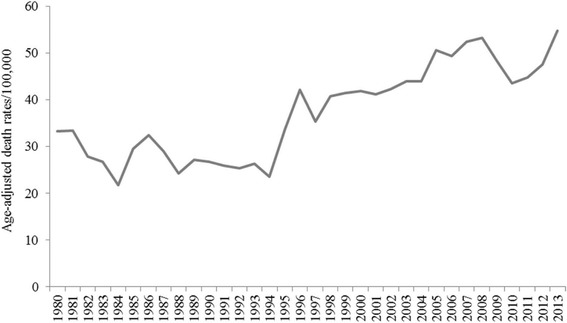
Trends in age-adjusted hypertensive heart disease mortality rates among Mississippians 35 years and older, 1980–2013

**Fig. 2:**
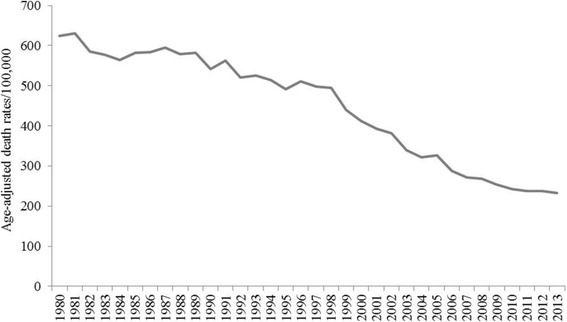
Trends in age-adjusted coronary heart disease mortality rates among Mississippians 35 years and older, 1980–2013

**Fig. 3:**
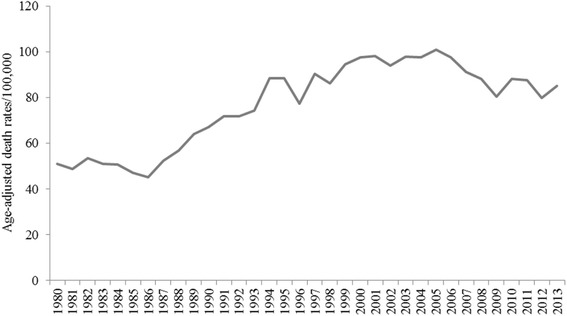
Trends in age-adjusted heart failure mortality rates among Mississippians 35 years and older, 1980–2013

Heart failure mortality rates also increased significantly among certain subgroups, although a different set, with an AAPC of 1.8% (95% CI 0.6 to 2.9) for women, 1.7% (95% CI 0.6 to 2.8) for whites, and 2.1% (95% CI 0.9 to 3.3) for individuals age 75 and older (Table 1, Figs. 1, 2 and 3).

**Table 1:** Trends in Mortality Rates among Mississippi Adults 35 years and older by Heart Disease Subtype, 1980–2013

				Sex		Race		Age Group, years		
HD Subtype^a^	Trend	Year	Total	Men	Woman	White	Black	35–54	55–74	≥75
Hypertensive	Deaths, No. (rate/100,000)^b^	1980	350	156	194	155	195	19	146	185
			(33.2)	(35.5)	(31.3)	(22.2)	(58.2)	(3.9)	(36.9)	(168.0)
		2013	864	410	454	450	410	112	282	470
			(54.8)	(59.4)	(50.0)	(39.5)	(93.9)	(15.0)	(47.1)	(270.8)
	AAPC (95% CI)		1.6	1.6^c^	1.5	2.0	1.2	4.5^c^	0.9	1.4^c^
			(−1.3,4.5)	(0.3,3.0)	(−1.0,4.0)	(−0.7,4.7)	(−1.8,4.4)	(3.6,5.5)	(−0.5,2.2)	(0.2,2.6)
Coronary Heart Disease	Deaths, No. (rate/100,000)	1980	6561	3565	2996	4569	1983	470	2704	3387
			(624.0)	(810.6)	(482.7)	(637.0)	(600.6)	(95.9)	(683.7)	(3075.1)
		2013	3750	2200	1550	2674	1050	441	1507	1802
			(232.5)	(313.1)	(168.0)	(230.8)	(227.7)	(59.0)	(251.9)	(1038.3)
	AAPC (95% CI)		−3.0^c^	−3.0^c^	−3.1^c^	−3.1^c^	−3.1^c^	−1.5^c^	−3.0^c^	−3.2^c^
			(−3.7,−2.3)	(−3.6,−2.3)	(−3.6,−2.5)	(−3.4,−2.7)	(−4.2,−2.0)	(−1.7,−1.2)	(−3.6,−2.3)	(−3.7,−2.8)
Heart Failure	Deaths, No. (rate/100,000)	1980	521	260	261	314	207	43	166	312
			(51.1)	(62.6)	(43.1)	(45.4)	(65.0)	(8.8)	(42.0)	(283.3)
		2013	1323	573	750	975	342	51	296	976
			(85.2)	(88.3)	(81.8)	(84.6)	(82.2)	(6.8)	(49.5)	(562.4)
	AAPC (95% CI)		1.4^c^	1.0	1.8^c^	1.7^c^	0.5	0.1	−0.1	2.1^c^
			(0.5,2.3)	(0.0,2.0)	(0.6,2.9)	(0.6,2.8)	(−1.1,2.2)	(−1.6,1.9)	(−1.6,1.3)	(0.9,3.3)

AAPC, average annual percent change; CI, confidence interval

^a^The table provides the international Classification of Diseases (ICD), Ninth and Tenth Revisions, for each HD Subtype

^b^Age adjusted using the 2000 U.S. standard population

^c^The AAPC is significantly different from zero

Coronary Heart Disease, ICD-9: 402, 410–414, 429.2 and ICD-10:I20-I25

Hypertensive HD, ICD-9:402 and ICD-10: I11, I13

Heart Failure, ICD-9:428 and ICD-10:I50

## Discussion

Our analysis of mortality rates for subtypes of HD among Mississippians age 35 and older from 1980 to 2013 produced mixed results. Overall, the CHD mortality rate declined significantly, while the heart failure mortality rate increased significantly. The significant decline in CHD mortality in Mississippi mirrors findings from the Atherosclerosis Risk in Communities (ARIC) study, which includes the city of Jackson, Mississippi [14], as well as the findings of a recent national study [7]. However, we observed a significant increase in heart failure mortality among Mississippians, which differs from the national trend [7]. Although HD mortality rates have declined in recent years in Mississippi [15], HD remains the leading cause of death, with 8962 average deaths annually [3]. The results indicated that the decline in HD mortality in Mississippi was mainly due to a significant decrease in CHD mortality across all race, sex, and age subgroups. National declines in HD morality have been attributed to primary HD prevention, early diagnosis, and improved treatment [5, 6, 16–19].

The magnitude of decline in CHD mortality was comparable across all sex, race, and age subgroups, with the exception of individuals age 35–54, whose magnitude of decline was half that of the overall group. The continual decline in HD mortality in Mississippi can be largely attributed to the dramatic decline in CHD mortality among all Mississippians age 35 and older. The ubiquitous decline in CHD may be due to increased risk factor awareness, better control of risk factors, early diagnosis, and improved treatment [5, 16–22]. Specifically, in a study of major risk factors in the 18-county Mississippi Delta region (a high cardiovascular disease burden region), we previously found that the prevalence of current smoking decreased significantly among white adults between 2001 and 2010 [23].

HD deaths attributable to hypertensive HD increased significantly among men, individuals age 35 to 54, and individuals age 75 and older during this focal period; the largest magnitude of increase occurred among those 35 to 54 years old. These increases may be due to an increasing prevalence of obesity and diabetes [24]. In 2013, Mississippi had the highest prevalence of adult obesity (35.1%) and second highest diabetes prevalence (12.9%) [25]. In addition, Mississippi adults have poor cardiovascular health [26, 27].

For heart failure mortality, we observed significant increases among women, whites, and individuals age 75 and older. Heart failure mortality increases among these populations may be due to an increase in the number of adults aged 75 and older, and a higher prevalence of obesity and diabetes [5, 28]. For example, the proportion of Mississippians 65 years and older grew from 11.5% in 1980 to 13.9% in 2013 [3], and in the 18-county Mississippi Delta region (the northwestern portion of the state) the prevalence of obesity and diabetes increased significantly from 2001 to 2010 [23].

These findings have potential limitations. First, reliance on death certificates may introduce bias due to the misclassification of either the primary cause of death [29, 30] or the decedent’s race [31], which can impact the conclusions drawn from epidemical studies [32]. However, death certificates are the only data source currently available to assess population trends in heart disease mortality (although they can be problematic if their accuracy varies over time) [30], and they allow the description of patterns in the entire population rather than a sample [33]. Second, changes in coding from ICD-9 to ICD-10 may affect the quality of death certificate data; however, a previous study validated the comparability of ICD-9 and ICD-10 in the analysis of mortality trends [34]. Finally, findings from a study of CHD deaths in New York City hospitals showed that CHD was over-reported as a cause of death on death certificates [35]; if this also occurred in Mississippi hospitals, the CHD mortality rates calculated in the current study would be biased. The extensive period of study and the analysis of population subgroups are key strengths of the study.

## Conclusions

In conclusion, from 1980 through 2013, overall CHD mortality rates experienced significant annual declines, while heart failure mortality increased significantly among Mississippi adults age 35 and older. The significant decline in deaths due to CHD observed in all race, sex, and age subgroups in Mississippi during this period is noteworthy. Hypertensive HD mortality increased significantly among men, those 35 to 54 years old, and individuals age 75 and older, while heart failure mortality increased significantly for whites, women, and individuals age 75 and older.

Ongoing state public health actions to prevent and control diabetes, heart disease, obesity and associated risk factors and promote school health in partnership in local stakeholders include interventions that seek to improve health, including reducing death and disabilities due to heart disease (www.cdc.gov/chronicdisease/about/state-public-health-actions.htm) [36]. In addition, in the 18-county Mississippi Delta region, the Mississippi State Department of Health, through the Mississippi Delta Health Collaborative (MDHC) and in collaboration with the Centers for Disease Control and Prevention (CDC) and local stakeholders, is currently implementing programs that aim to prevent and reduce heart disease, stroke, and associated risk factors, with a focus on the ABCS (aspirin for those at risk, blood pressure control, cholesterol management, and smoking cessation) of heart disease and stroke prevention (www.healthyms.com/MDHC).

## Abbreviations

AAPC: Average annual percent change
CHD: Coronary heart disease
CI: Confidence interval
HD: Heart disease
ICD: International Classification of Diseases
MDHC: Mississippi Delta Health Collaborative
